# Acute Posterior Multifocal Placoid Pigment Epitheliopathy: Clinical and Iconographical Aspects of Two Cases

**DOI:** 10.7759/cureus.88485

**Published:** 2025-07-22

**Authors:** Zakaria Azemour, Nabil Bouslous, Ahmed R Idihia, Moulahid Loubna, Moustaine M Omar

**Affiliations:** 1 Ophthalmology Department, Souss-Massa University Hospital, Agadir, MAR

**Keywords:** apmppe, multimodal ophthalmic imaging, papillitis, serous retinal detachment, white dot syndrome

## Abstract

Acute posterior multifocal placoid pigment epitheliopathy (APMPPE) is a rare inflammatory disorder of the choriocapillaris and retinal pigment epithelium. We report two atypical cases: one with unilateral involvement complicated by serous retinal detachment requiring corticosteroid therapy; the second with bilateral involvement that resolved spontaneously. These observations underscore the clinical heterogeneity of APMPPE and the pivotal role of multimodal imaging, including optical coherence tomography (OCT) and OCT-angiography (OCT-A), in elucidating its pathophysiology, guiding diagnosis, monitoring disease progression, and tailoring treatment strategies.

## Introduction

Acute posterior multifocal placoid pigment epitheliopathy (APMPPE) is a rare inflammatory disease that primarily affects the choroid and retinal pigment epithelium (RPE). Originally described by Gass in 1968 [1], APMPPE typically presents in young adults, often after a viral-like prodrome or systemic inflammatory condition. The disorder manifests with sudden visual impairment, photopsia, and scotoma due to multiple yellow-white placoid lesions in the posterior pole of the retina [2]. While the condition is often self-limiting, it can sometimes lead to significant ocular and systemic complications.

The pathophysiology of APMPPE remains unclear; however, recent advances in multimodal imaging have provided significant insights into its underlying mechanisms. Current evidence suggests that the inflammatory process primarily affects the choriocapillaris and extends into the RPE and outer retina [3].

APMPPE may appear to be a benign, self-limiting condition with a favorable prognosis; however, in cases of systemic involvement, it can lead to serious complications such as cerebral vasculitis, ischemic stroke, and meningoencephalitis with severe visual outcomes [2]. These complications are thought to be linked to a vasculitis process that disrupts the blood-retinal barrier, leading to ischemic damage in both ocular and extraocular tissues [4].

This report describes two unusual cases of APMPPE with rare clinical presentations characterized by the association of disc edema (papillitis) and serous retinal detachment (SRD): the first case was unilateral, whereas the second was bilateral.

## Case presentation

Case one

A 36-year-old female with no significant medical history, with no history of recent vaccinations or flu-like symptoms, presented with a rapidly progressive decrease in visual acuity (VA) in her left eye (LE), that had begun 10 days prior to her admission, accompanied by left-sided headaches and mono paresthesia in her left upper limb, occurring five days before admission.

**Figure 1:**
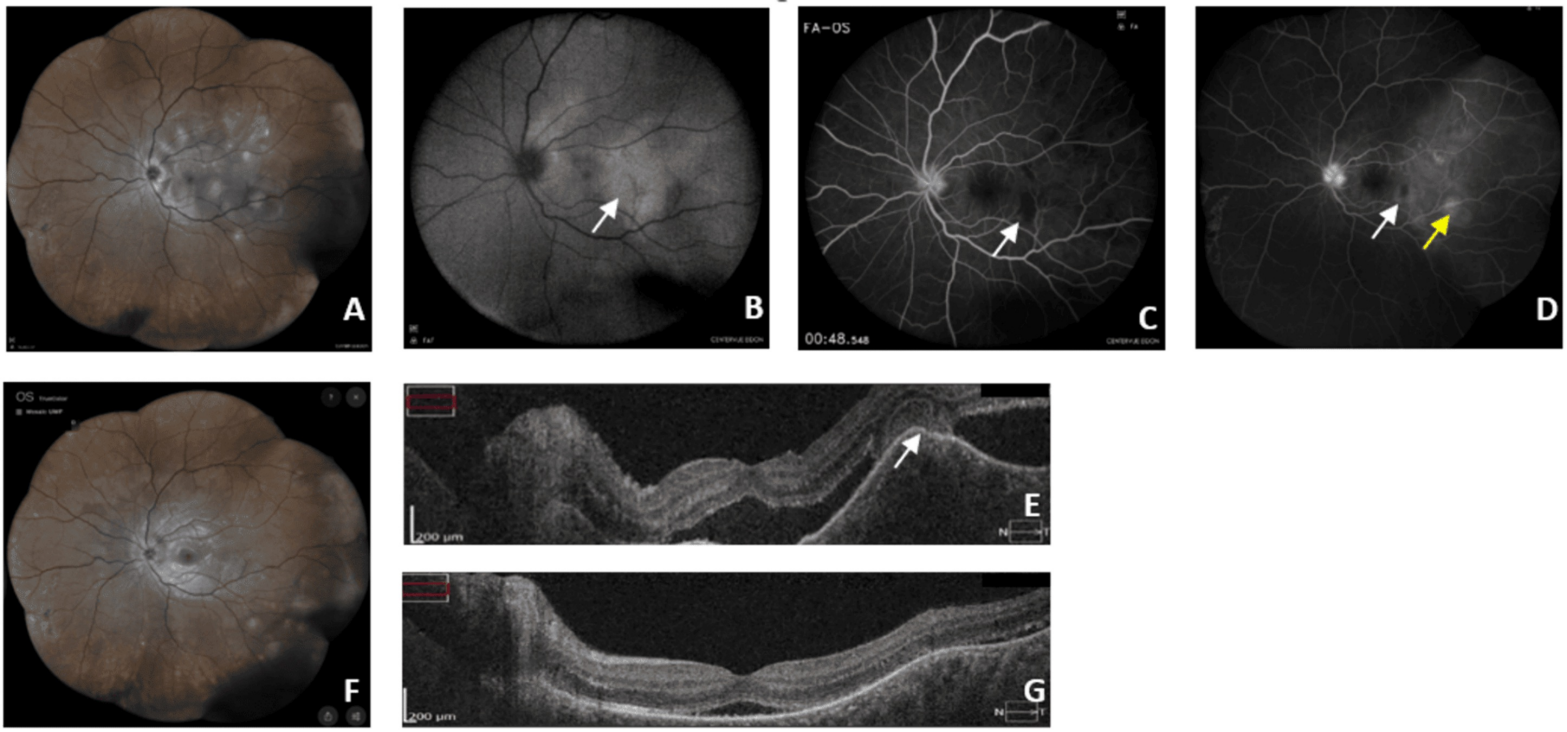
Fundus photography, FAF, FA and OCT of right eye of the first case A) Retinophotography showing multiple white-yellowish placoid lesions at the posterior pole in the level of the RPE; B) FAF showing areas of hyperautofluorescence (white arrow); C) Early retinal FA showing hypofluorescent lesions (white arrow); D) Late retinal FA showing hypofluorescent lesions surrounded by hyperfluorescence (white arrow) and hyperfluorescent lesions (yellow arrow); E) OCT revealed a SRD, outer retinal disruption and presence of hyperreflective area (white arrow) corresponding to placoid lesion; F) Retinophotography showing significant regression of lesions five weeks after; G) Control OCT showing regression of SRD five weeks after. FAF - fundus autofluorescence; FA - fluorescein angiography; OCT - optical coherence tomography; RPE - retinal pigment epithelium; SRD - serous retinal detachment

At the initial examination, the best-corrected visual acuity (BCVA) in the LE was counting fingers at 1.5 meters. The Intraocular pressure (IOP) and anterior segment examination were normal without any sign of ocular inflammation. Fundus examination revealed multiple white-yellowish placoid lesions at the posterior pole in the level of the retinal pigment epithelium (RPE) and choroid, with blurred borders, along with papillitis, without Relative afferent pupillary defect (RAPD), vitreous haze, or signs of vasculitis (Figure 1A). The right eye (RE) examination was normal, with a BCVA of 10/10.

Fundus autofluorescence (FAF) of the left eye (LE) showed areas of hyper-autofluorescence (Figure 1B). The fluorescein angiography (FA) demonstrated in early-phase hypo-fluorescent lesions corresponding to the placoid foci, which became hyperfluorescent sometimes with a central hypo-fluorescence in the late phase (Figure 1C-D).

Macular optical coherence tomography (OCT) revealed a serous retinal detachment in the LE, outer retinal disruption, and presence of placoid lesions (Figure 1E), with a normal aspect of the RE.

The neurological examination was normal, except for headaches and paresthesia in the left upper limb. Brain and orbital MRI were normal. A comprehensive biological workup, including a complete blood count (CBC), erythrocyte sedimentation rate (ESR), C-reactive protein (CRP), Treponema pallidum hemagglutination assay (TPHA), Venereal Disease Research Laboratory (VDRL) test, angiotensin-converting enzyme (ACE) level, and tuberculin skin test, was likewise unremarkable.

Oral prednisolone (60 mg /day) was initiated. At day 15, ophthalmologic examination showed favorable progression, with improvement in visual acuity to 8/10, reduction of retinal lesions, and resolution of headaches and paresthesia. Following significant clinical improvement, corticosteroid tapering was initiated, with complete discontinuation at one month. A follow-up ophthalmological examination after stopping corticosteroid therapy showed, visual acuity to 10/10 and an almost complete resolution of fundoscopic lesions, re-attachment of the retina with a minimal residual SRD (Figure 1F).

Case two

A 22-year-old woman with no significant medical history was admitted for decreased vision in the left eye (LE) seven days ago. The patient had already experienced an episode of decreased VA in her RE with no other associated symptoms 20 days ago, with a spontaneous total resolution.

Her ophthalmological examination has shown a VA at 10/10 in the RE but is limited in her LE at counting fingers. Anterior segment examination was normal in both eyes. Indeed, fundoscopy revealed multiple well-defined, non-confluent yellowish-white placoid lesions at the posterior pole extending to the equatorial region. Additionally, optic disc edema was observed: grade 3 in the RE and grade 1 in the LE without RAPD. 

**Figure 2:**
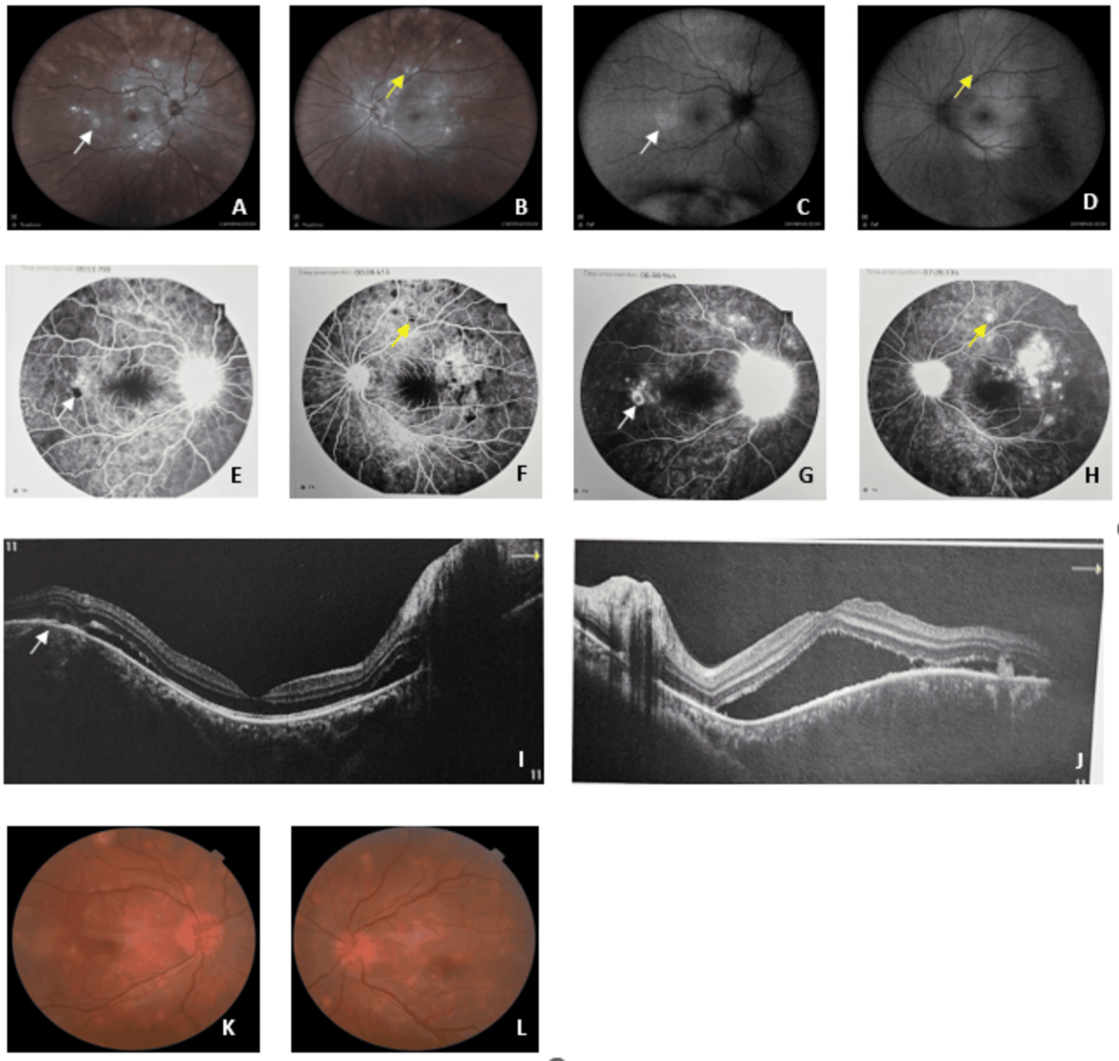
Fundus photography, FAF, FA and OCT of the second case (the white indicator shows the same lesion of the right eye through different types of multimodal imaging, while the yellow indicator shows the same lesion of the left eye.) A) Retinophotography of the RE showing multiple white-yellowish placoid lesions at the posterior pole in the level of the RPE and extending to the equatorial region; B) Retinophotography of the LE showing multiple white-yellowish placoid lesions at the posterior pole in the level of the RPE and extending to the equatorial region; C) FAF: Hyper-autofluorescence of RE in location of placoid lesion; D) FAF: Hyper-autofluorescence of LE in location of placoid lesion; E) FA: Early hypo-fuorescence in the locations of placoid lesions in the RE; F) FA: Early hypo-fuorescence in the locations of placoid lesions in the LE; G) FA: Late hyper-fuorescence in the locations of placoid lesions in the RE; H) FA: Late hyper-fluorescence in the locations of placoid lesions in the LE; I) OCT showing a small SRD, retinal thinning in location of placoid lesion, disruption and hyperreflective of outer retina in RE; J) OCT showing in SRD, disruption and hyperreflective of outer retina LE; K) Retinophotography of RE showing significant regression of placoid lesions four weeks after; L) Retinophotography of LE showing significant regression of placoid lesions four weeks after FAF - fundus autofluorescence; FA - fluorescein angiography; OCT - optical coherence tomography; RE - right eye; LE - left eye; RPE - retinal pigment epithelium; SRD - serous retinal detachment

FAF revealed hyper-autofluorescence at the location of the placoid lesions (Figure 2C, D). Early phase fluorescein angiography showed hypo-fluorescent lesions at the level of the outer retina and choroid, that turned later hyper-fluorescent, sometimes centered by hypo-fluorescence with staining of the papilla (Figure 2H, G). Macular OCT of the RE showed a minimal SRD with disruption of the outer retina; OCT of the LE showed an SRD involving the macular region, with disruption and hyper-reflectivity of the outer retina (Figure 2I, J).

The neurological examination was normal. The biological workup, including CBC, ESR, CRP, TPHA, VDRL, tuberculin skin test, and ACE, was also within normal limits. A watchful waiting approach was adopted due to the absence of clinical signs suggestive of systemic involvement. The evolution was marked by a complete recovery of VA to 10/10 within one month.

## Discussion

Acute posterior multifocal placoid pigment epitheliopathy (APMPPE) is a rare inflammatory disease that primarily affects the choroid and retinal pigment epithelium (RPE). It was originally described by Gass in 1968 [1].

In our cases, APMPPE was presented atypically. The first form was unilateral (case one), while the second one was bilateral and asymmetric (case two). Clinically, there was a sudden decrease in visual acuity, multiple yellowish-white placoid lesions at the posterior pole, papillary involvement, and an SRD detected on OCT. These features underscore the heterogeneous ways in which APMPPE can present and highlight the importance of prompt multimodal evaluation.

From a pathophysiological standpoint, APMPPE is considered an inflammatory entity primarily targeting the choroid and extending to the RPE and photoreceptors [2]. Choriocapillaris inflammation compromises choroidal perfusion, leading to ischemic lesions and RPE dysfunction. The placoid lesions seen on fundus examination, together with early hypo-fluorescence and late hyper-fluorescence on FA, reflect these ischemic and inflammatory processes [5]. In our cases, multimodal imaging (FA, OCT, and autofluorescence) was pivotal in confirming the posterior location of these lesions.

Although APMPPE is often regarded as benign and self-limiting, some patients develop ocular or systemic complications, such as optic neuropathy, macular edema, or cerebral vasculitis leading to ischemic events [2]. In the first case presented, the unilateral disease was accompanied by prominent papillitis and an SRD, highlighting a locally severe inflammatory process. However, no neurological involvement was detected by MRI, and rapid improvement under oral corticosteroids, which was initiated due to the suspicion of systemic involvement, prompted by unilateral headaches and mono-paresthesia in the left upper limb, with a favorable outcome. In the second case, observation alone led to complete spontaneous resolution, illustrating the variability in disease severity and management strategies ranging from simple monitoring to high-dose corticosteroids [2].

Therapeutic decisions in APMPPE usually depend on the severity of visual impairment and extraocular involvement. In mild forms, spontaneous remission is often seen and can justify regular monitoring [2]. Conversely, severe presentations, particularly those affecting the macula or accompanied by neurological complications, may require systemic corticosteroids or other immunosuppressants to control inflammation and prevent permanent damage [2]. In the first patient, rapid functional recovery was achieved with early steroid therapy; in contrast, the second patient's bilateral form resolved without pharmacological intervention. These contrasting outcomes emphasize the heterogeneous clinical spectrum of APMPPE and the need for individualized treatment, guided by close follow-up and imaging.

APMPPE should be distinguished from other white-dot syndromes such as serpiginous choroiditis, multiple evanescent white dot syndrome (MEWDS), or birdshot chorioretinopathy. However, the distinctively placoid appearance, the angiographic findings, and significant RPE involvement usually differentiate APMPPE from these entities [2]. Vogt-Koyanagi-Harada (VKH) syndrome was ruled out as it did not meet the diagnostic criteria, and other infectious causes were excluded by a negative infectious workup.

Recent data obtained through OCT-angiography (OCT-A) have helped delineate the course of APMPPE lesions over time more precisely. Burke et al. propose a four-stage evolutionary sequence, starting from an exclusively choroidal insult to a full-fledged chorioretinal involvement, followed by a transitional phase and eventual resolution [5]. In the earliest stage (phase 1), OCT-A reveals significantly altered choroidal perfusion even when conventional OCT or FAF appear normal [5]. As lesions "progress" (phase 2), hyper-reflective changes in the outer retinal layers become apparent on OCT, along with the typical hypo- and hyper-fluorescence patterns on FA [5]. During the transitional phase (phase 3), the abnormalities persist but begin to regress centrifugally, with partial choroidal reperfusion and gradual RPE remodeling [5]. Finally, the last phase (phase 4) shows normalization of the choriocapillaris signal on OCT-A in most patients, though mild structural sequelae may remain (outer retinal thinning, subtle RPE disruptions) and a residual hypo-autofluorescence appearance can be seen [5]. This phase-based description underscores the value of OCT-A for detecting vascular changes at a very early stage and for following the course of lesion recovery or progression in a non-invasive manner. It also highlights the clinical heterogeneity of APMPPE, while some lesions can regress without leaving any trace, others will cause more pronounced and lasting tissue alterations.

With corticosteroid therapy, the first case rapidly progressed to the resolved phase (stage 4), with full visual recovery and near-complete disappearance of the lesions, leaving only minor pigmentary scarring at the RPE level. The second, which had an asynchronous bilateral presentation, demonstrated a spontaneously favorable course: the right eye had already reached the resolved stage by the time of evaluation (normal acuity with only subtle RPE pigmentary changes), while the left eye was in an active chorioretinal phase at diagnosis and subsequently passed through a transitional phase of partial lesion regression to ultimately achieve resolution within a few weeks. These cases thus align with the Burke et al. model, supporting the notion that APMPPE begins as a reversible ischemic choriocapillaritis that secondarily induces outer retinal/RPE damage, which then heals with residual RPE pigmentary alterations

## Conclusions

These two cases illustrate the clinical variability of APMPPE, ranging from a unilateral form requiring systemic steroids to a bilateral but spontaneously resolving form. Timely diagnosis is critical for guiding therapy, especially when systemic complications are suspected.

New imaging modalities such as OCT-A play an essential role in detecting early choroidal hypoperfusion, monitoring disease progression, and refining treatment strategies. Future research, including larger studies and longer follow-up, may clarify prognosis factors and help optimize patient care.
